# Quantitative Analysis of Salivary Oral Bacteria Associated with Severe Early Childhood Caries and Construction of Caries Assessment Model

**DOI:** 10.1038/s41598-020-63222-1

**Published:** 2020-04-14

**Authors:** Lijuan Zhang, Tongzheng Sun, Pengfei Zhu, Zheng Sun, Shanshan Li, Fan Li, Ying Zhang, Kaixuan Tan, Jie Lu, Rongtao Yuan, Zhenggang Chen, Dawei Guo, Qingyuan Guo, Fei Teng, Fang Yang

**Affiliations:** 1 School of Stomatology, Qingdao University, Qingdao, Shandong 266003 China; 2 Stomatology Center, Qingdao Municipal Hospital, Qingdao, Shandong 266071 China; 3 Department of Stomatology, the Ninth People’s Hospital of Qingdao, Qingdao, Shandong 266071 China; 4 Single-Cell Center, Qingdao Institute of Bioenergy and Bioprocess Technology, Chinese Academy of Sciences, Qingdao, Shandong 266101 China

**Keywords:** Microbiology, Medical research

## Abstract

To construct a saliva-based caries risk assessment model, saliva samples from 176 severe early childhood caries (S-ECC) children and 178 healthy (H) children were screened by real-time PCR-based quantification of the selected species, including *Streptococcus mutans*, *Prevotella pallens*, *Prevotella denticola* and *Lactobacillus fermentum*. Host factors including caries status, dmft indices, age, gender, and geographic origin were assessed in their influence on abundance of the targeted species, which revealed host caries status as the dominant factor, followed by dmft indices (both P < 0.01). Moreover, levels of *S*. *mutans* and *P*. *denticola* in the S-ECC group were significantly higher than those in the healthy group (P < 0.001 for *S*. *mutans* and P < 0.01 for *P*. *denticola*). Interestingly, the co-occurrence network of these targeted species in the S-ECC group differed from that from the healthy group. Finally, based on the combined change pattern of *S*. *mutans* and *P*. *pallens*, we constructed an S-ECC diagnosis model with an accuracy of 72%. This saliva-based caries diagnosis model is of potential value for circumstances where sampling dental plague is difficult.

## Introduction

Early childhood caries (ECC) is defined as the presence of one or more decayed, missing, or filled tooth surfaces in the primary dentition in children of 71 months or younger^1^. Severe early childhood caries (S-ECC), an extraordinary form of ECC, is defined as the presence of decayed, missing, or filled score surfaces of either ≥4 (age 3 years), ≥5 (age 4 years), or ≥6 (age 5 years)^2^. In USA, 23% of children between the ages of 2 and 5 are affected by ECC^3^. In China, fresh reports from the Fourth National Oral Health Survey showed that over 70% of 5-year-old children carry dental caries in primary teeth^4^. Unfortunately, childhood caries are wide-ranging, rapid-progressing and irreversible^5^. Besides, severe caries can cause pulpal infection, as well as varieties of adverse physical and psychological effects, thus it affects children’s development while posing a substantial economic burden on both families and society^6–8^. Therefore, preventive measures and early diagnosis of ECC or S-ECC are of vital clinical and social importance.

Many studies have shown that caries is a multifactorial disease^9,10^ and pathogenic bacteria are the main cause of disease occurrence and progression^11^. *Streptococcus mutans* (*S*. *mutans*) has been considered as a cariogenic bacterial agent in children^12–15^, due to its aciduric and acidogenic properties^16^. Apart from this, *Lactobacillus* spp. was also linked to caries development and progression^16–18^. Positive associations between certain *Lactobacillus* spp. (especially *Lactobacillus fermentum*) and the hard tissue changes were revealed in the process of caries progression^19,20^. In addition, our past pyrosequencing of oral and plaque microbiota unveiled *Prevotella* spp’s close relationship with caries, in both cross-sectional and longitudinal studies^21,22^. Specifically, we proposed a caries risk assessment model based on the relative abundance of seven *Prevotella* spp. (*Prevotella pallens*, *P*. *denticola*, *P*. *verovalis*, *P*. *salivae*, *P*. *histicola*, *P*. *DO039* and *P*. *maculosa*), which features 74% accuracy in predicting new onsets of ECC^22^. A number of studies have also reported strong association between several *Prevotella* spp. (in particular *P*. *denticola*) and caries status as well^23–25^.

However, at present it is not clear whether the absolute amount of above caries-associated taxa showed differential features among S-ECC and healthy; and moreover, if we could construct a saliva based efficient and economic caries diagnosis model based on PCR quantification. Therefore here we designed a cross-sectional study of 354 children, including 176 caries-active children (dmft≥6) and 178 healthy children (dmft=0), and quantify caries-associated organisms including *S*. *mutans*, *L*. *fermentum*, *P*. *pallens*, and *P*. *denticola* from saliva via quantitative real-time PCR (qPCR).

## Results

### Quantifying absolute abundance of selected bacteria from saliva

**Figure 1:**
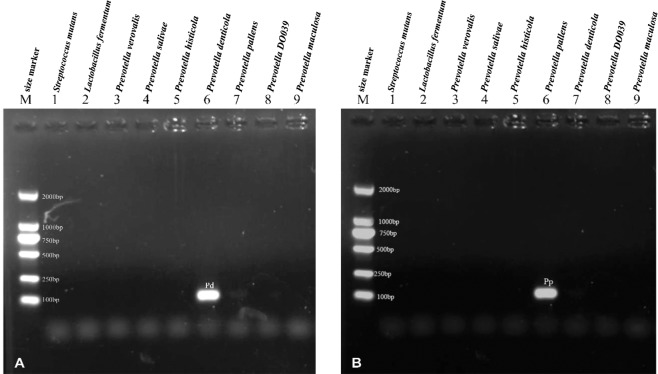
Verification of the specificity of those two *Prevotella* primers. (**A**) Specificity of the primer pair targeting *P*. *denticola*. Lane 1–9 showed the amplification result of *P*. *denticola* primer pair from the genomic DNA of various oral species. Amplification was positive only when using *P*. *denticola* DNA as template. (**B**) Specificity of the primer pair targeting *P*. *pallens*. Lane 1–9 showed the amplification result of *P*. *pallens* primer pair from the genomic DNA of various oral species. Amplification was positive only when using *P*. *pallens* DNA.

In total, 354 children (3–5 years of age), including 176 severe early childhood caries (S-ECC) (dmft≥6) children and 178 healthy (H) (dmft=0) children, were screened saliva sample collection. Detection and quantification of the selected species in salivary samples were performed by qPCR. The absolute amounts of *S*. *mutans*, *P*. *pallens*, *P*. *denticola* and *L*. *fermentum* were assessed by specific qPCR primers. Two pairs of primers (for *S*. *mutans* and *L*. *fermentum*) were used based on the published primer protocols (Table 1). Another two pairs of new primers (for *P*. *pallens* and *P*. *denticola*) were designed using AlleleID 6.0 (Premier Biosoft, Palo Alto, CA, USA) for qPCR and then analyzed in BLASTn (https://blast.ncbi.nlm.nih.gov/Blast.cgi?PAGcE_TYPE=BlastSearch). Specificity of above newly designed primers were verified in Fig. 1. The result showed that both the *P*. *denticola* and *P*. *pallens* primer pairs displayed good specificity at the species level. The thermal conditions of qPCR reactions were listed in Table 2.

**Table 1:** DNA primers used in the qPCR analysis.

Target species	Primer name	Primer sequence (5′ → 3′)	Amplicon Size(bp)	References
*Prevotella denticola*	Pd-R-12	AGATGGATGCAGAGCTGAAGC	150	—
	Pd-F-12	GTCACCGACCTGGATCTTACG		
*Prevotella pallens*	Pp-16SB-F	AGCCTGAACCAGCCAAGTAG	150	—
	Pp-16SB-R	GCCGGTCCTTATTCATACGATAC		
*Streptococcus mutans*	Sm-F2	GCAGTCAAGGGGTGGAAATCG	188	^38^
	Sm-R2	TGGACGGCTTGTTGCAGGAATAC		
*Lactobacillus fermentum*	LF-F	TGGAAACAGRTGCTAATACCG	231–233	^19^
	LF-R	GTCCATTGTGGAAGATTCCC

**Table 2:** Thermal cycling conditions for qPCR analysis.

Species	Thermal cycling conditions				
	Pre-heating	denaturation	annealing	elongation	cycles
*Prevotella denticola*	94 °C, 10 min	94 °C, 15 s	60 °C, 20 s	72 °C, 15 s	30
*Prevotella pallens*	94 °C, 10 min	94 °C, 15 s	60 °C, 20 s	72 °C, 15 s	30
*Streptococcus mutans*	94 °C, 10 min	95 °C, 10 s	62–72 °C, 30 s	70 °C, 30 s	30
*Lactobacillus fermentum*	95 °C, 10 min	95 °C, 15 s	62 °C, 1 min	50 °C, 2 min	30

### Influence of hosts’ factors in defining species levels

To compare the effect sizes of the various host factors on the bacterial levels, permutational multivariate analysis of variance (PERMANOVA) was applied. As shown in Table 3, among the five hosts’ factors including age, gender, caries-status, dmft indices and geographical origin, caries status displayed the strongest effect in defining the bacterial level, followed by dmft indices (F = 43.757, F = 3.420, respectively, both P < 0.01). No significant effect was observed from hosts’ age, gender, and geographic origin on the level of these species (P > 0.05). Hosts’ geographic origin also contributed to the differential load of those species, in which the amounts of *S*. *mutans* and *P*. *pallens* from northern city were significantly higher than those from the southern city (all P < 0.001, Mann-Whitney’s U test). However, no significant difference was found within these species’ levels among genders (P > 0.05, Mann-Whitney’s U test).

**Table 3:** Influence of host factors on the abundance of selected species.

	F value	*P* value
Caries status	43.757	1.06E-03**
The dmft indices	3.420	1.14E-03**
Age	1.818	1.65E-01
Gender	0.537	4.46E-01
Geographic origin	0.086	6.81E-01

P < 0.05 was considered statistically significant. The asterisks denoted statistical significance (**P < 0.01).

### Comparisons of the levels of specific species between healthy and caries-active children

Levels of those four species were compared between the S-ECC and healthy groups (Table 4). *P*. *denticola* and *S*. *mutans* in the S-ECC group were significantly more abundant than those in the healthy group (P < 0.01 and P < 0.001, respectively, Mann-Whitney’s U test). In contrast, no significant difference was found between the healthy and S-ECC groups for *P*. *pallens* and *L*. *fermentum* (P > 0.05). In addition, no significant difference was found within those species’ level among genders (P > 0.05, Mann-Whitney’s U test) (Table 4).

**Table 4:** The absolute abundance of target species in saliva as measured by qPCR.

		*Streptococcus mutans*	*Prevotella pallens*	*Prevotella denticola*	*Lactobacillus fermentum*
**Healthy and S-ECC groups**					
**Healthy**	mean	4.02E + 04	1.06E + 03	2.83E + 05	1.23E + 00
	SD	2.40E + 02	3.74E + 01	8.43 E + 02	1.34E + 00
**S-ECC**	mean	1.10E + 06	1.45E + 03	3.82E + 05	2.84E + 00
	SD	1.12E + 03	4.06E + 01	1.01E + 03	2.00E + 00
	P value (Wilcoxon.test)	1.88E-28***	1.34E-01	9.66E-03**	5.51E-02
**Southern and northern city groups**					
**Southern city**	mean	9.25E + 04	3.55E + 02	1.85E + 05	1.41E + 00
	SD	4.39E + 02	2.20E + 01	9.68E + 02	1.26E + 00
**Northern city**	mean	4.69E + 05	1.90E + 03	3.95E + 05	2.65E + 00
	SD	2.14E + 06	9.63E + 03	9.11E + 02	1.98E + 00
	P value (Wilcoxon.test)	3.62E-04***	4.60E-16***	2.16E-01	4.77E-01
**Male and female groups**					
**Male**	mean	7.53E + 05	1.15E + 03	3.47E + 04	1.81E + 00
	SD	1.39E + 03	3.72E + 01	2.80E + 02	1.63E + 00
**Female**	mean	7.63E + 05	1.51E + 03	3.26E + 04	2.15E + 00
	SD	1.43E + 03	4.45E + 01	2.92E + 02	1.76E + 00
	P value (Wilcoxon.test)	6.77E-01	6.77E-01	9.47E-01	6.77E-01

The values shown were represented by scientific notation. The units of the means were copies/μL. The red arrows indicate elevated trend. S-ECC: severe early childhood caries; SD: standard deviation; Mann-Whitney’s U test was used for statistical analysis. P < 0.05 was considered statistically significant. Asterisks denoted statistical significance (**P < 0.01; ***P < 0.001).

### Correlation between species level and dmft indices

To understand the correlation between the selected species and dmft indices, we collected the hosts’ caries status data including number of decayed, missing and filled tooth from each individual and calculated the dmft indices. A strong positive correlation between *S*. *mutans* level and dmft indices was found (Table 5; Spearman’s rank correlation coefficient r = 0.600, P < 0.001). No significant correlation between other species levels and dmft indices was observed.

**Table 5:** Correlation between the levels of targeted species in saliva and the dmft index scores.

	Spearman’s rank correlation coefficient	*P* value
*Streptococcus mutans*	0.600	2.17E-08 ***
*Prevotella denticola*	0.142	1.42E-01
*Lactobacillus fermentum*	0.125	2.89E-01
*Prevotella pallens*	0.070	1.00E + 00

P < 0.05 was considered statistically significant. The asterisks denoted statistical significance (***P < 0.001).

### The co-occurrence networks of the targeted species in caries and health

**Figure 2:**
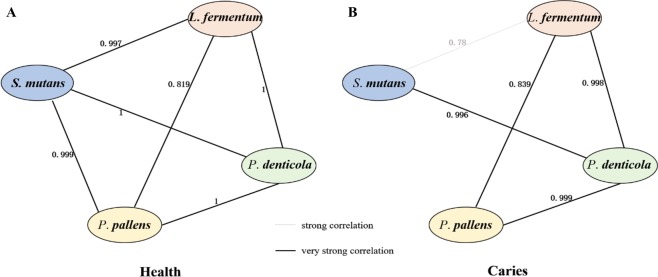
The co-occurrence networks of the targeted species in each of the two hosts groups. The connection lines between two nodes indicate positive correlation between the levels of two species, with color representing the degree of correlation. There was a very strong positive correlation among every species in a pair-wise manner in the healthy group (**A**). In the S-ECC group, however, the correlation between *S*. *mutans* and *L*. *fermentum* was weakened, and the correlation between *S*. *mutans* and *P*. *pallens* was no longer present (**B**).

The co-occurrence network can reveal the ecological relationship between the bacterial species in the microbial community. In the healthy group, there was a very strong positive correlation among the levels of each strains, including *S*. *mutans*, *L*. *fermentum*, *P*. *pallens*, and *P*. *denticola* (Fig. 2A). However, the correlation pattern changed in the S-ECC group (Fig. 2B). Specifically, the correlation between *S*. *mutans* and *L*. *fermentum* was weakened (the coefficient value changed from 0.997 to 0.78), and correlation between *S*. *mutans* and *P*. *pallens* disappeared in the S-ECC group (the coefficient value changed from 0.999 to 0.103). This result suggested that the salivary community structures were differed in disease state, and the weaken or even disappeared connection among the tested members in caries samples indicated a diversely distributed community structure.

### Building a diagnosis model of S-ECC using *P. pallens* and *S. mutans*

**Figure 3:**
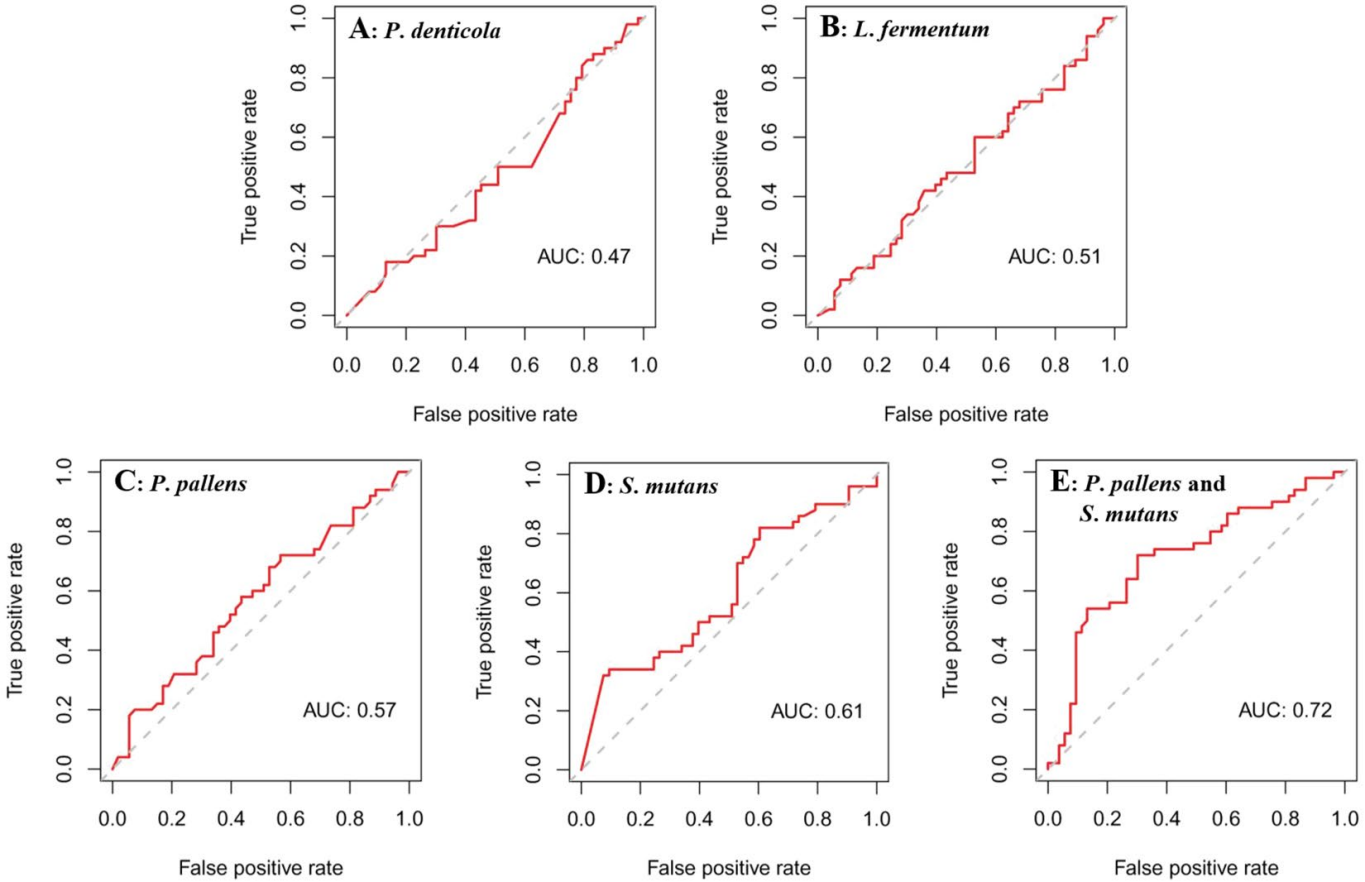
ROC curves of the caries classification models. The AUC values of models derived from *P*. *denticola* alone (0.47; (**A**)), *L*. *fermentum* alone (0.51; (**B**)), *P*. *pallens* alone (0.57; (**C**)), or from *S*. *mutans* alone (0.61; (**D**)). (**E**) The model was built from combining *P. pallens* and *S. mutans*. It carries an AUC of 0.72, higher than the single-species models.

To probe which of the four species exerts the greatest effect on model performance, a series of models were built from every singular species. The AUC values of models derived from the sole species of *P*. *denticola*, *L*. *fermentum*, *P*. *pallens* or *S*. *mutans* were 0.47, 0.51, 0.57 and 0.61, respectively (Fig. 3A–D). According to “rfcv” function in the Random-Forest package, the two top-ranking significant taxa (*P*. *pallens* and *S*. *mutans*) from these selected species led to a reasonably good classification of S-ECC status. And the two-species model built from *P*. *pallens* and *S*. *mutans* showed a relatively higher predictive power to distinguish S-ECC from the healthy groups (AUC = 0.72; shown in Fig. 3E). Specifically, through testing absolute amount of *P*. *pallens* and *S*. *mutans*, we could differentiate those hosts with severe caries disease with the accuracy of 72%.

## Discussion

In this study, we aimed to utilize qPCR technology, a more simpler, cost-effective and time-saving method for accurate, sensitive and rapid quantification of those selected species in the salivary microbiome^26,27^. We found that the level of *S*. *mutans* in S-ECC was significantly higher than those from the healthy group (P < 0.001), which was consistent with the previous studies^18,28^. The dysbiosis of the oral microbiome, such as *S*. *mutans*, from an overproduction of acid, can result in increasing proportions of acidogenic and aciduric species^29^. However, no significant difference was found on the level of *L*. *fermentum* among the healthy and S-ECC groups in our study, which was also a recognized acidogenic caries pathogen. In contrast, another article reported that the levels of *Lactobacillus* spp. in plaques were significantly elevated in children with severe ECC patients^18,30^. Above conflicting results might be attributed to different sampling methods. Specifically, our study was based on saliva samples, while previous studies have used carious dentin samples. It indicated that *L*. *fermentum* might contribute more in the frontier of dentin caries, however, saliva-based quantification of *L*. *fermentum* could not detect significant difference in patients.

Our previous study tracked the changes of microbiota over time from the healthy status to caries occurrence and caries progression, thus developed a model for caries prediction and suggested a panel of *Prevotella* species that may be closely related to caries disease^21,22^. *Prevotella*’s association with caries was also verified by many other studies^24,25^. These indicated that the overexpressed collagenases for proteolytic metabolism in *Prevotella* species may lead to the progression of dental caries^23^. Therefore, the design of this study involved *P*. *denticola* and *P*. *pallens*, two of the seven most discriminant *Prevotella* species in the prediction and diagnosis model for ECC^22^. Although above two *Prevotella* spp. both contribute to great extent in caries risk prediction, only *P*. *denticola* was detected with significantly elevated absolute amount in S-ECC group (P < 0.01) by real-time PCR-based quantification, the amounts of *P*. *pallens* were nearly the same between the groups. This result suggested that the amount of the species might not directly linked to its effect on disease prediction model construction.

Caries status and dmft indices were the top two factors in defining the absolute abundance of those selected species, yet factors like age, gender and geographic origin did not influence the bacteria levels significantly. This suggested that even the individuals were from different background, disease status could still discriminate the S-ECC community structures from H groups. Interestingly, in healthy children, there were very strong positive correlations between two of the four targeted strains. In contrast, in children with S-ECC, the very strong positive correlation between *S*. *mutans* and *L*. *fermentum* found in the healthy children was significantly weakened, and the very strong positive correlation between *S*. *mutans* and *P*. *denticola* found in the healthy children was even disappeared. This finding was consistent to our former result that healthy microbiomes were more conversed, while those caries microbiomes were more diversely distributed^21,22^. For the healthy group, as they were more resembled, so we were able to detect more consistence in the close relationship of those bacterial members among this group. However, for the caries group, a shifted balance of microbiota takes place in the oral environment^21,22,31^, where any bacterial members with the ability of acid-producing and acid-resisting could potentially initiate the occurrence of caries. This might be a potential explanation for that on the links between the levels of chose bacterial members especially between those acknowledged caries-leading bacteria like *S*. *mutans* and *L*. *fermentum*, *S*. *mutans* and *P*. *pallens* were weaken or disappeared in the S-ECC group.

In terms of caries diagnosis assessment model, neither single species could elicit a satisfied diagnostic power with AUC from 0.47–0.61, even the significantly differentially distributed *P*. *denticola* resulted in the lowest accuracy of 0.47. However, the combination of *S*. *mutans* and *P*. *pallens* results in a caries assessment model with an accuracy of 72%, which was nearly equal to our former ECC prediction model (74% accuracy) based on eight marker *Prevotella* species via pyrosequencing^22^. In this study, based on a cross-sectional experimental design, we aimed to monitor levels of specific potential caries-associated bacterial markers and evaluate their contribution to caries diagnosis model construction. What deserve our attention is, levels of *P*. *denticola* (not *P*. *pallens*) were significantly higher in S-ECC, but the model finally constructed was derived from *P*. *pallens* and *S*. *mutans*. This suggested that the abundance of species might not be the sole predictor for caries^32^ and links among species can be exploited to discriminate caries status in the models. In addition, our result indicated that *S*. *mutans* played a significant role in the caries diagnosis model, and a model that combined *S*. *mutans* and *P*. *pallens* reached accuracy of 72%. However, the caries prediction model we built before were composed of a panel of seven *Prevotella* species with accuracy of 74%^22^, and *S*. *mutans* didn’t contribute to this model. This indicated that there was difference in dominant pathogens during caries onset and caries progression.

In this study, utilizing the rapid, accurate and economic qPCR technique, we developed a saliva-based efficient and economic S-ECC risk assessment model. Traditional methods of diagnosing ECC include visual-tactile detection combining with bitewing radiography. In addition, radiography, transillumination, ECM device, and methods based on fluorescence are useful for caries detection^33^. However, all these methods necessitate a certain extent of children’s cooperation and on-site at the dental chair, and they can also be time-consuming and laborious^33,34^. Therefore, the caries diagnosis model built here can be beneficial to preschool age children, especially for those children who are anxious and thus unable to cooperate for oral exams, and can also be adopted for remote screening or home-based survey of caries risk for epidemiological studies.

## Materials and methods

### Selection of subjects for this study

The children employed in this study were from an oral health census (June 2017) in kindergartens at the southern city of Guangzhou (the Guangdong Province) and the northern city of Qingdao (the Shandong Province), which are physically separated by two thousand kilometers in mainland China. After an oral health survey, 354 children (3–5 years of age), including 190 boys and 164 girls, were chosen for saliva sample collection. All children were unrelated individuals of both genders^21^. According to the number of dental caries assessed with a decayed, missing, filled tooth (dmft) indices, 176 children were classified as S-ECC (dmft ≥ 6) and 178 children were classified as healthy (dmft = 0). All the guardians of the children were made aware of the nature of the experiment and granted written permission for participation. The written permission and study design had been approved by the Ethical Committee of Qingdao University (Qingdao, China). All experiments were performed following relevant guidelines and regulations. No child wore a removable appliance or took antibiotics in the preceding three months. Children with systematic or other oral diseases such as mucosal diseases and/or were excluded^21^.

### Sample collection and DNA extraction

The clinical examinations and assessments of caries, as well as salivary sample collection, were carried out by dentists who were previously trained for the assessments of caries and sampling procedures. Unstimulated whole saliva (2 mL) was collected from each child into a tube containing an equal volume of lysis buffer (50 mM EDTA, 50 mM sucrose, 50 mM Tris, pH 8.0, 100 mM NaCl and 1% SDS)^35^. Salivary samples were stored at −80 °C before DNA extraction^21^. The extraction of DNA from bacterial cultures was performed using an optimized protocol based on the Qiagen DNeasy Blood & Tissue DNA kit (QIAGEN, Hilden, Germany) according to the manufacturer’s instructions. DNA concentrations were determined using a Qubit Fluorometer 2.0 (Life Technologies, Grand Island, NY, USA). The purity of the extracted DNA was measured by the Qubit dsDNA HS Assay Kit (Invitrogen, Carlsbad, California, USA) following the manufacturer’s instructions, with an inclusion criterion of above 1.8. Electrophoresis of DNA was performed to assess DNA integrity under ultraviolet light. The extracted DNA samples were stored at −80 °C before further processing.

### Design of quantitative qPCR primers

Detection and quantification of the selected species in salivary samples were performed by qPCR. The presence of *S*. *mutans*, *P*. *pallens*, *P*. *denticola* and *L*. *fermentum* was detected by specific qPCR primers. Two pairs of primers (for *S*. *mutans* and *L*. *fermentum*) were used based on the published primer protocols (Table 1). Another two pairs of new primers (for *P*. *pallens* and *P*. *denticola*) were designed using AlleleID 6.0 (Premier Biosoft, Palo Alto, CA, USA) for qPCR and then analyzed in BLASTn (https://blast.ncbi.nlm.nih.gov/Blast.cgi?PAGcE_TYPE=BlastSearch)^1^. Species specificity for the primers of *P*. *denticola* and *P*. *pallens* were tested by conventional **(**Fig. 1**)**. The thermal conditions of qPCR reactions were listed in Table 2.

### Quantitative real-time PCR

Each reaction mixture (20 μL) was composed of 10 μL of SYBR Green Master Mix, 0.5 μL of each forward/reverse primer (10 μM), 5 μL of sterilized DNase-RNase-free water, and 4 μL of DNA sample. The qPCR reaction was performed in Microamp fast optical 96-well reaction plates (Applied Biosystems, Foster City, CA, USA) using a LightCycler 480II (Roche, Basle, Switzerland). The qPCR reaction of samples was performed in triplicate and a negative control (ddH_2_O as a template) was included within each experiment^36^. Standard curves of primers were obtained by measuring five 10-fold series diluted DNA standards (Targeted DNA fragment cloned in plasmid pMD19T)^37^. Reaction specificities were confirmed via melting curve analysis with a progressive increase in temperature and continuous fluorescence acquisition. The standard DNA amplification curve and melting-point product curve for each primer combination were obtained to calculate the quantity of DNA.

### Statistical analysis

Statistical analyses were performed using R software (@Manual {, title = {igraph: Easily Install and Load the ‘igraph’}, author ={Patrick R. Amestoy}, organization ={AMD library}, address ={California, American}, year = 2019, url ={https://CRAN.R-project.org/package=igraph}}). Mann-Whitney U test was applied to the quantitative data of salivary microbiome. P < 0.05 was considered as the threshold for statistical significance for all tests. Asterisks were used to denote statistical significance (*: P < 0.05; **: P < 0.01; ***: P < 0.001). Association of the selected species levels and dmft indices, as well as the co-occurrence networks of the targeted species, were estimated using the Spearman correlation coefficient.

### Construction of risk assessment model for S-ECC

Firstly, the Random Forests method was employed to discriminate between diseased and healthy subjects from the southern city cohort. The receiver operating characteristic (ROC) curve was used to evaluate the diagnostic value of bacterial candidates in discrimination between diseased and healthy subjects. According to “rfcv” function in the Random-Forest package, the two top-ranking significant taxa from these selected species led to a reasonably good classification of ECC status. Model performance was then assessed using a 10-fold cross-validation approach^22^. Secondly, the southern city cohort was used as a training dataset and the northern city cohort was used as a testing dataset to evaluate the discriminatory power of the model, which was further evaluated using the area under the ROC curve (AUC).
